# A practical guide to sequencing in neuropsychiatric research

**DOI:** 10.1038/s44277-025-00041-0

**Published:** 2025-08-08

**Authors:** Adelaide R. Minerva, Michael DeBerardine, Rixing Lin, Hye Ji J. Kim, Forrest D. Rogers, Rebekah L. Rashford, Catherine J. Peña

**Affiliations:** Princeton Neuroscience Institute, Princeton University, Princeton, NJ 08544 USA

**Keywords:** Genome informatics, Sequencing

## Abstract

Technology and computational analysis to profile RNA and DNA at genome-wide scale has rapidly evolved in the last two decades, with a concomitant rise in their application within neuroscience and psychiatry research. These technologies initially offered “unbiased” insights and the potential to discover previously unconsidered molecular mediators of disease and development. The more recent advent and adoption of single-cell/nucleus and spatial “omics” sequencing provides unprecedented insights into cellular processes within heterogeneous tissues. These advances are especially advantageous in neuropsychiatric research, which faces unique challenges due to the brain’s cellular heterogeneity, dynamic development, and the complex, polygenic nature of many psychiatric disorders. Still, different sequencing techniques are better suited for different questions and the most fine-grained (and expensive) approaches are not always necessary. Here, we offer a simple primer on the pros, cons, and best applications for currently available sequencing technologies.

## Lay summary

This review offers a simple overview on the pros, cons, and best applications for currently available genome-wide sequencing technologies in neuropsychiatric research.

## Introduction

Since it became commercially available in the 2000s, next generation sequencing (NGS) has revolutionized nearly every area of biomedical research. NGS refers to a suite of high-throughput technologies capable of sequencing millions of genetic molecules in parallel, dramatically lowering costs and enabling large-scale analysis. Early consortium-led studies, such as the Human Genome Project [1], focused on whole genome sequencing (WGS) and produced the first large-scale genome assemblies. These efforts laid the foundation for a growing ecosystem of sequencing approaches, including epigenome and transcriptome profiling. A major innovation was the use of reverse transcription to convert RNA into sequenceable DNA, resulting in the now ubiquitous RNA-seq. Over time, researchers began using NGS not only to determine nucleotide sequences, but as a general readout for biological phenomena. Additional chemical or enzymatic selection steps now allow for the creation of libraries that reflect chromatin accessibility, DNA-protein interactions, chemical modifications, and different RNA classes, giving rise to the dizzying array of sequencing-based methods available to researchers today.

NGS technologies are especially powerful in neuropsychiatric research, where the brain’s cellular and spatial diversity, layered developmental programs, and high degree of inter-individual variability create unique challenges. Bulk sequencing offers useful but averaged gene expression that may obscure subtle or cell-type-specific differences. Single-cell sequencing can resolve measurements at the cellular level, enabling the classification of diverse neuronal and glial subtypes and detection of small but potentially meaningful molecular changes relevant to disease. When combined with spatial or perturbation approaches, NGS can offer unprecedented insights into the genetic and epigenetic basis of disease, neurodevelopment, and drug or treatment response.

**Fig. 1:**
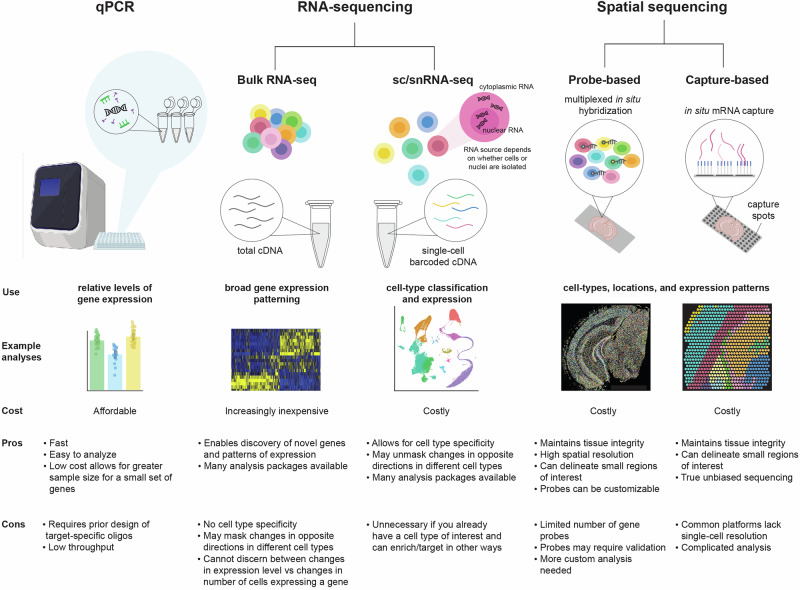
Comparisons of targeted and genome-wide RNA profiling assays.

Despite their transformative potential, sequencing technologies vary in resolution, cost, and the most high-resolution (and costly) method may not always be best suited for different research questions. Here, we give a brief overview of the most commonly used sequencing technologies, their best applications, and practical considerations for experimental design and analysis. We also include references to application of each approach in neuropsychiatric research (Fig. 1).

### Targeted gene analysis: semi-quantitative polymerase chain reaction (qPCR)

Real-time semi-quantitative qPCR measures relative abundance of RNAs (converted to cDNA) from a tissue sample, such that expression is at a “bulk tissue” level across all cells in the sample. qPCR uses pairs of primers to amplify specific genes of interest. Depending on starting quantity of purified RNA and reaction preparation, expression of dozens of genes can be measured (with separate primer pairs) and compared across samples and groups. qPCR is sensitive, cost effective, and efficient. However, because it requires a known sequence for genes of interest, it is best suited for studies with a small number of hypothesis-driven gene targets. In addition, qPCR requires careful primer design to ensure specificity, efficiency, and avoid off-target amplification. qPCR is also used to validate findings from high-throughput sequencing methods. Despite the widespread adoption of NGS methods, qPCR remains a powerful and trusted tool for well-designed, hypothesis driven questions in neuropsychiatric research.

### RNA-sequencing

RNA-seq measures gene expression across the entire transcriptome and therefore allows for gene agnostic, hypothesis-free measurements. RNA-seq involves converting RNA to complementary DNA (cDNA), fragmenting it, and “reading” the order of nucleotides with serial imaging of complementary fluorescent bases. These sequences can then be aligned to known genomes to determine which genes are present and at what level. While early RNA-seq used Sanger sequencing [2], NGS [3] is now typically the method of choice, due to its high throughput, sensitivity, speed, and lower cost.

#### Single-cell RNA-seq

In recent years, single-cell methods have revolutionized RNA-seq by allowing nucleotide sequences to be associated with individual cells through the use of cellular barcoding. Traditional RNA-seq involves preparation of a merged RNA-seq library from all cells in a sample (“bulk”), where the resulting library measures average gene expression. While this can be useful for identifying broad trends, it is a major limitation for tissues with high degrees of cellular heterogeneity like the brain.

A common approach for single-cell library preparation uses a microfluidic device to separate individual cells or nuclei into droplets that contain unique barcode sequences. These droplet-specific barcodes allow for RNA-seq reads to be grouped together into individual cells of origin during analysis. The most typical and practical downstream result of this is that major cell types (e.g., neurons and glia) can be grouped together and analyzed separately. This is essential for understanding cellular heterogeneity [4, 5], how cells interact [6–8], or how diverse cell types are altered differently across conditions [9–12].

While often referred to generically as “single-cell” methods, there is a distinction between methods that isolate whole cells or nuclei for library preparation. The use of whole cells in scRNA-seq better captures the overall transcriptome of a cell (as most mRNA is found in the cytoplasm), whereas nuclear isolation (snRNA-seq) enriches for newly transcribed RNA that has yet to be exported to the cytoplasm. Thus, snRNA-seq has increased sensitivity to dynamic changes in transcription during development or in response to stimuli. Despite these differences, nuclei provide comparable gene expression information to whole cells [13, 14]. In the case of brain tissue, snRNA-seq generally prevails due to a number of practical considerations, particularly (1) the ease and much greater recovery of isolated nuclei as opposed to dissociated cells due to the shearing of processes [15, 16], (2) better preservation of neuronal RNA, (3) reduced cell type bias, as some cell types dissociate more easily [17, 18], (4) minimized artificial activation of immediate early genes (IEGs), and (5) better ability of nuclei to flow through microfluidic sorting devices.

While single-cell approaches are very commonly used for RNA-seq experiments, various other sequencing methods have been adapted to single-cell, some of which are discussed below.

#### Long-read RNA-sequencing

While short-read sequencing maintains an overwhelming advantage in throughput and cost-per-read, long-read sequencing has key advantages for specific applications. The ability to sequence full-length RNA transcripts allows for entire transcript isoforms to be characterized (i.e., the entire set of introns and exons present in individual transcripts) and long genomic reads are essential for generating modern genome assemblies. This is especially valuable for characterizing splicing abnormalities like intron retention, which are implicated in neurodegenerative and psychiatric diseases [19–21].

Nanopore sequencing [22, 23], a unique long-read approach, directly sequences long DNA or RNA molecules in real time. This is achieved by passing a nucleic acid molecule through a tiny protein nanopore embedded in a membrane. As each nucleotide moves through the pore, it causes distinct changes in electrical current that are measured and translated into a RNA sequence. While historically limited by higher error rates compared to short-read sequencing, recent advancements in base calling algorithms and improved sequencing modes have significantly increased the raw read accuracy, making it a powerful tool for many applications such as characterizing transcriptomic complexity of the brain at the isoform level.

Recent years have also seen the development of long-read scRNA-seq [24]. While still relatively uncommon, long-read scRNA-seq has been used to identify extensive and conserved RNA isoform variation during brain development [25], and that complex RNA isoform usage is predictive of ASD, intellectual disability, and neurodegenerative disorders [26].

#### Spatial RNA-sequencing

The sequencing methods described above use homogenized tissue as input and thereby lose information about where cells are located and how they are organized within the tissue. Spatial sequencing complements traditional methods by providing spatial context, which can be essential for many questions of biology such as how cell types covary across space [27–29] or how anatomically distinct cell populations respond differently to injury or disease [30, 31]. Preservation of the location of gene expression within intact tissue is especially valuable in the brain, where anatomical organization is linked to function and pathology.

Spatial sequencing comes in two forms: probe-based profiling methods like MERFISH [32] or 10X’s Xenium platform [33] and true genome-wide sequencing-based methods like Slide-Seq [34] or 10X’s Visium platform [35]. The former are essentially multiplexed fluorescent *in situ* hybridization experiments, where a preselected set of probes is iteratively hybridized, imaged, and washed off from a tissue slice. This technology is quickly advancing to allow for the use of greater numbers of probes (from hundreds to now thousands), but may require custom probe design if predesigned probe panels do not suffice. True genome-wide spatial sequencing, in contrast, uses spatially barcoded slides that are able to capture RNA from fresh frozen tissue. The tissue is imaged before being fixed and permeabilized, releasing RNA to bind to nearby capture probes. Later, the spatial barcodes allow for individual RNA-seq reads to be assigned to coordinates on the slide (called “spots”) and spatially placed within the imaged tissue. While this approach allows for genome-wide detection, the “spots” contain reads from multiple cells–in one estimate, an average of 3.3 cells per spot in brain tissue [36]–and so the resolution is typically not truly single-cell. This requires an additional deconvolution step to resolve cases where spots contain disparate cell types. By contrast, probe-based spatial sequencing methods allow for even subcellular spatial resolution.

### DNA sequencing

In its most basic application, NGS enables the large-scale observation of human genetic variation and identification of genetic variants. Unlike earlier microarray-based methods, NGS allows for the discovery of *de novo* mutations and rare variants that may contribute to disease. This shift has been reflected in resources like dbSNP and ClinVar, which increasingly incorporate sequencing-derived data over older array-based entries. In large-scale genome-wide association studies (GWAS), disease-linked variants can be identified, which in turn implicate specific genes and gene-regulatory elements in pathology. These links are often critical components in the design or analysis of the kinds of sequence-based experiments discussed in this review.

### Epigenetic sequencing

Chromatin is broadly divided into two groups: closed, inaccessible heterochromatin (which is generally unavailable for functional interactions), and open, accessible euchromatin. Transcribed genes and the cis-regulatory elements that modulate their expression (e.g., enhancers and repressors) are all found within euchromatin. The accessibility of chromatin plays a key role in determining which genes can be expressed in a given context. Within euchromatin, there are varying degrees of accessibility and numerous chromatin states, which can be identified by their diverse post-translational histone modifications [37]. Some of the most fundamental insights addressed by epigenetic sequencing methods are the identification of cis-regulatory elements, which proteins bind them, and which genes those elements and proteins regulate. Identifying these gene regulatory relationships is essential for understanding the molecular mechanisms of disease.

#### ATAC-seq

The assay for transposase-accessible chromatin followed by sequencing (ATAC-seq) is a widely used method for identifying accessible regions of chromatin, making it incredibly useful for identifying cis-regulatory elements. ATAC-seq was first introduced in 2013 and required the use of fresh tissue [38] but has since been optimized for use with frozen tissue, broadening its utility in both preclinical and clinical research.

ATAC-seq uses the Tn5 (transposase) enzyme, which cuts DNA preferentially at sites of open chromatin. When preloaded with sequencing adapters, Tn5 simultaneously isolates accessible chromatin to fragment DNA and insert adapters, generating sequence-ready molecules in a process known as “tagmentation.” ATAC-seq library preparation is fast, cost-efficient, and has low background, largely replacing earlier methods for DNA accessibility profiling like DNase-seq and FAIRE-seq.

ATAC-seq has recently been adapted as a single-cell method [39, 40] and is often paired with scRNA-seq in single-cell “multiome” experiments that capture chromatin accessibility and gene expression from the same cells [41]. This integration allows for powerful insights into how gene regulation is coordinated within individual cells.

While ATAC-seq does not directly measure chromatin-protein interactions, motif analysis within accessible regions can be used alongside known transcription factor binding motifs to infer putative binding events. Particularly in the context of single-cell multiome experiments, the ability to correlate expression of transcription factors with accessibility of their putative binding sites and expression of genes near those binding sites provides a powerful basis to infer regulatory relationships between transcription factors and potential target genes [42].

Recent studies have demonstrated the utility of ATAC-seq in neuropsychiatric research. For example, by generating a chromatin accessibility atlas of the prefrontal cortex of healthy and schizophrenia patients, researchers identified over 100,000 accessible regions enriched for psychiatric disease associated variants [43]. In the fetal brain, ATAC-seq was used to map regulatory dynamics underlying cortical development and neurodevelopmental disorder risk [44].

#### ChIP-seq, Cut&Run, Cut&Tag

While ATAC-seq identifies regions of open chromatin, antibody-based methods allow for direct physical measurement of protein-DNA interactions, offering a more detailed view of chromatin architecture than accessibility profiling alone by targeting specific transcription factors or histone modifications. Commonly used methods include ChIP-seq (Chromatin-immunoprecipitation), CUT&RUN (“Cleavage Under Targets and Release Using Nuclease”), and CUT&TAG (“Cleavage Under Targets and Tagmentation”), all of which share a common requirement for validated antibodies, but differ in their input requirements, chromatin fragmentation method, antibody/target compatibility, sequencing capacity, workflow time, and cost efficiency.

In classic ChIP [45, 46], cells undergo cross-linking fixation before being lysed to release chromatin, which is then fragmented by sonication. Antibodies are used to immunoprecipitate protein-bound DNA fragments from solution, which are then sequenced. The size of fragmentation determines the specificity of protein-DNA complex localization (e.g., if chromatin is fragmented to ~250 bp of DNA, the protein of interest may be bound anywhere in that fragment), although in practice the resolution is somewhat higher as numerous fragments will overlap to generate a peak. Major drawbacks of ChIP-seq are its generally high input requirements and high background, which together make it less cost efficient. Additionally, while some highly stable protein-DNA complexes can forgo the crosslinking step (“Native ChIP”), the general requirement for formaldehyde cross-linking can itself sometimes destroy transient protein-DNA interactions [47] and only “ChIP-grade” antibodies (which tolerate cross-linking) are compatible.

Rather than lysing cells and fragmenting bulk chromatin, CUT&RUN [48, 49] directly binds micrococcal nuclease (MNase) to the antibody being used. When the antibody binds its DNA-bound protein target, MNase cleaves chromatin directly “under the target”, releasing small fragments of DNA which are easily isolated and sequenced. CUT&RUN requires lower sample input and is less labor intensive than classic ChIP, has higher resolution and reduced background, is more cost effective, and does not strictly require ChIP grade antibodies due to the absence of formaldehyde cross-linking. However, in certain cases (like with large molecular complexes), the conjugated enzymes can be physically unable to reach around the complex to cut chromatin.

CUT&Tag is very similar to CUT&RUN, but uses Tn5 in place of MNase [50, 51]. This allows rapid library preparation using the same “tagmentation” reaction used in ATAC-seq. However, CUT&Tag can require more optimization and troubleshooting than CUT&RUN.

#### DNA-methylation sequencing

DNA methylation is a crucial epigenetic modification involving the enzymatic addition of methyl groups to the DNA molecule. The most common and well-characterized type, 5-methylcytosine (5mC), predominantly occurs at cytosines preceding guanines (CpG) dinucleotides [52, 53]. 5mC can directly influence gene expression by methylating promoter regions, thereby preventing the binding of transcription factors and repressing gene activity [54]. The genomic pattern of 5mC is highly dynamic throughout development and is frequently dysregulated in disease states [55].

Bisulfite sequencing (BS) has served as the gold standard for comprehensive DNA 5mC analysis. This method leverages the chemical treatment of DNA with sodium bisulfite, which selectively deaminates unmethylated cytosines to uracil while leaving methylated cytosines unaffected [53, 56]. Subsequent PCR amplification converts uracil to thymine, enabling the differentiation between methylated and unmethylated cytosines after sequencing and comparing to untreated DNA sequence. While whole-genome bisulfite sequencing (WGBS) offers single-base resolution methylation maps and comprehensive genomic coverage, it necessitates substantial sequencing depth and consequently incurs high costs. To address this cost barrier, reduced representation bisulfite sequencing (RRBS) was developed [53, 57]. RRBS enriches for CpG-rich regions, such as CpG islands, using restriction enzymes prior to bisulfite conversion, providing a more cost-effective alternative for profiling methylation within specific genomic contexts. A significant limitation of sodium bisulfite treatment, however, is its potential to cause DNA damage and degradation, leading to over-fragmentation and sample loss. Furthermore, bisulfite treatment alone cannot distinguish between 5mC and 5-hydroxymethylcytosine (5hmC), an oxidized form of 5mC, rendering it impractical when both modifications require investigation [58].

To overcome the specificity limitation, antibody-based methods like methylated DNA immunoprecipitation sequencing (MeDIP-Seq) and DNA immunoprecipitation sequencing (DIP-seq) were developed [59, 60]. Following a similar concept to ChIP-seq, these methods involve incubating fragmented genomic DNA with specific anti-5mC antibodies, followed by immunoprecipitation, library construction, and sequencing. A major drawback of these antibody-based approaches is their inability to provide single-base resolution. Instead, they identify “clusters” or “peaks” of 5mC modifications but oftentimes require less sequencing costs [59, 60].

More recently, methods such as enzymatic methyl-sequencing (EM-seq) have emerged. EM-seq relies on enzymatic conversion of cytosine to uracil, significantly minimizing DNA damage and enabling successful library preparation from lower DNA input amounts. This method also allows for the continued application of existing bioinformatics analysis pipelines developed for BS or RRBS data [61]. Additionally, commercially available EM-seq kits have integrated steps for specific detection of 5mC and 5hmC. The insights gained from DNA methylation sequencing are paramount for advancing our understanding of gene regulation, developmental processes, and the pathogenesis of a wide range of diseases.

### RNA modifications and interactions

#### CLIP-seq

While ChIP-seq uses antibodies to immunoprecipitate (IP) fragments of chromatin for sequencing, CLIP-seq (Crosslinking and Immunoprecipitation followed by sequencing) combines IP and RNA sequencing to identify sites where RNA-binding proteins (RBPs) bind RNA [62]. This can reveal how RBPs influence processes like splicing, stability, and translation. In one study [63], CLIP-seq was used to identify binding sites of RBPs implicated in alternative splicing and Autism Spectrum Disorders (ASD), revealing that dozens of their RNA targets were genes previously implicated in ASD. This study demonstrates how CLIP-seq can uncover molecular mechanisms linking RNA-protein interactions to complex disorders and highlights the role of post-transcriptional regulation in neurodevelopment and disease.

#### MeRIP-seq

Methylated RNA Immunoprecipitation Sequencing (MeRIP-seq) [64] is a method similar to CLIP-seq, but instead of mapping RNA-protein interactions, it uses IP to identify and quantify N6-methyladenosine (m6A), the most common chemical modification found on mRNA. m6A is critical for gene regulation, with key roles in RNA stability, splicing, and translation. A recent study using MeRIP-seq [65] found that patient-derived cells with repeat expansion in C9ORF72 (the most common genetic cause of ALS and frontotemporal dementia) had globally reduced m6A levels. These changes were implicated in aberrant gene expression patterns potentially associated with neurodegeneration. This study highlights the importance of RNA modifications in disease and the power of MeRIP-seq to uncover disruptions that may not be visible by RNA abundance alone.

### 3D genomic architecture profiling

While the epigenetic/chromatin-profiling methods discussed above can identify putative gene enhancers, it can be difficult to confidently determine which genes those enhancers may be acting on. To address this, chromatin conformation capture technologies are used to study the three-dimensional organization of the genome by identifying genomic loci that are physically close to one another. If two distant loci (e.g., a putative enhancer and a gene promoter) are physically close to one another in 3D space, this suggests a functional interaction between them. These methods rely on a technique called “proximity ligation,” which involves crosslink fixation of nuclei to preserve its structure, followed by digestion of DNA into smaller pieces and re-ligation of the broken DNA ends together. If loci are able to ligate to one another, that demonstrates that they were physically close.

#### Targeted chromatin capture methods

The original 3 C method (Chromosome Conformation Capture) uses PCR with specific primers to two sites of interest [66]. This is a “one-to-one” assay in which potentially-interacting loci are assayed one pair of sites at a time. 3C was extended to “one-to-many” in the 4C method (Circular Chromosome Conformation Capture), which targets only one of locus for analysis, allowing for unbiased detection of all of the loci that interact with it [67]. 5C (Carbon Copy Chromosome Conformation Capture) enables a “many-to-many” approach to identify all interactions within a given ~1 Mb genomic region of interest [68], allowing for more comprehensive interaction mapping.

#### Genome-wide chromatin interaction mapping

Chromatin capture was later scaled to be genome-wide (“all-vs-all”) in High-throughput Chromosome Conformation Capture (Hi-C) [69], which maps the overall 3D architecture of the genome. Hi-C has been used to study gene regulatory mechanisms in human brain development [70] and disease pathophysiology [71]. Micro-C is an enhanced version of Hi-C that uses micrococcal nuclease instead of restriction enzymes to create higher resolution maps [72]. While powerful, these methods require deep sequencing coverage to detect meaningful interactions and are therefore resource-intensive and expensive.

#### Targeted enrichment for functional interactions

Methods like promoter-capture Hi-C (PCHi-C) aim to overcome the cost and sensitivity limitations of Hi-C by enriching for informative interactions [73]. When combined with comprehensive probe sets, PCHi-C maintains almost “genome-wide” coverage [74]. This approach has been used to link non-coding genetic variants, such as those associated with schizophrenia and depression, to specific target genes, revealing potential mechanisms underlying these complex conditions [75].

### Experimental design, common analysis, and practical considerations

#### Power and replication

As with any experiment, the design of a sequencing study should align with the biological question. Sequencing experiments might include as few as one group (e.g., if the goal is characterization of cell types in a particular brain region), while groupwise comparisons require multiple biological replicates. As with other types of experiments, identifying effects associated with more subtle phenotypes or manipulations will require greater sample sizes. Determining sample size (N) necessary to properly power a sequencing study can be difficult and vary by type of assay [76] but tools have been built to address this question in bulk [77] and single-cell applications [78]. Isogenic animal models may require fewer biological replicates per group than human studies, although transcriptome-level sequencing requires fewer replicates than human SNP and GWAS studies. Generally, analysis has found that to detect 2-fold differences in gene expression in rodent studies, an N of 6-7 mice per group was required, but increasing sample size to an N of 8-12 significantly reduced false-positive rates [79]. In single-cell/nucleus experiments, sequencing a higher number of cells at a lower read depth gave greater power than sequencing fewer cells more deeply [78]. Notably, in single-cell studies, the N is the number of samples, not the number of cells, and analyses should be performed on pseudo-bulked data, as described below.

#### Sample quality control

No matter your technique of choice, sequencing quality depends on the integrity of your input sample. Poor sample quality compromises downstream analyses and interpretability (“garbage in, garbage out”) and should be identified early. Tools such as Nanodrop, Qubit, Bioanalyzer, or TapeStation should be used to assess RNA or DNA concentration and quality before sequencing. Input normalization should be used, and spike-in controls should be used if changes in the total signal are expected to differ across groups.

#### Cell-type identification

A major advantage of single-cell over bulk approaches is the ability to assign transcripts to individual cells. This is critical for studying complex tissues like the brain, where cellular heterogeneity is a defining feature. However, defining what constitutes a “cell type” remains complex and often context dependent [80, 81]. Frequently used approaches for cell type identification such as clustering and binning require thoughtful parameter selection and biological validation. In brain research, major consortium-led efforts are generating brain cell type atlases and hierarchical cell type taxonomies, which can provide a common reference and nomenclature for cell typing [5, 82]. Use of these cell type atlases is facilitated by tools like MapMyCells from the Allen Institute for Brain Science (RRID:SCR_024672).

#### Differential gene expression (DGE) analysis

Differential gene expression (DE or DGE) analysis is a generic term for the statistical frameworks and significance tests used for identifying genes that change across conditions. While the approach (and terminology) is typical of RNA-seq, the same software packages and basic statistical approaches are used for various “omics” methods e.g., ChIP-seq (“differential binding”) or ATAC-seq (“differential accessibility” or DA analysis). The most commonly used implementations of DGE analysis are DESeq2, edgeR, and limma-voom, and we strongly recommend the use of one of these packages [83–85]. Each of these frameworks offer valid but somewhat distinct solutions to the statistical problems of DGE analysis and are each widely accepted and their results are generally concordant [86, 87].

“Pseudobulking” single-cell data refers to summing and normalizing counts for each gene across all cells in a given cluster, as though each specific cell type was individually purified and bulk-sequenced. Pseudobulking is important because it properly treats the sample size as number of distinct samples rather than the number of cells sequenced. Pseudobulking allows for statistically robust conventional DGE analysis to be performed, but ignores any cellular heterogeneity within a cell type/cluster. While various methods have been developed for DGE analysis in single-cell datasets [88–91], pseudobulking remains the standard and robustly validated approach [92–94].

Once DGE is calculated, one can simply focus on most up- or down-regulated genes, although much more is possible. DGE is typically visualized with volcano plots (log of fold-change vs -log of p-value), MA plots (basal expression vs log of fold-change), or heat maps (visualization of degree of log-fold-change after application of a cut-off threshold for adjusted p-values and fold-change considered significant).

#### Enrichment analysis

Particularly in cases when many genes are identified as being differentially expressed, researchers often use annotation databases to identify whether these genes have known links to one another. Gene Set Enrichment Analysis (GSEA) is a common framework used to identify enrichment of genes according to Gene ontology (GO) term annotations, KEGG pathways, or other previously identified gene sets, which may reflect involvement in certain biological processes, functions, cellular localizations, or disease associations. Multiple comparisons can also be compared through joint heatmaps using traditional thresholded criteria, or in a threshold-free manner across the transcriptome such as by rank-rank hypergeometric overlap analysis [95] to give insight into patterns. Examples of many of these analyses applied to analyzing the impact of stress in bulk and single-cell sequencing can be found here [96–99].

##### Motif enrichment

Identification of short, overrepresented DNA or RNA sequences, termed motifs, near differentially expressed genes or within co-expression modules can point to upstream regulatory elements such as transcription factors that may be responsible for driving expression patterns in one’s dataset. This approach can be used on its own with motif databases such as JASPAR [100] or combined with ATAC-seq or ChIP-seq.

##### Network analysis

Multiple methods exist to describe the correlation patterns among groups of genes rather than individual genes and which do not rely on DGE. For example, weighted gene co-expression network analysis (WGCNA) identifies modules of highly co-expressed genes that can be linked to traits (e.g. disease status, age, brain region) or to hub genes that may act as central drivers of a module. Since many psychiatric disorders involve dysfunction of gene networks rather than isolated genes, analyzing co-expression modules can reveal broader and potentially more meaningful patterns of change across groups [9, 10, 96, 101]. WGCNA and related gene network analysis approaches are an important complement to DGE analysis because disease and manipulations may not whole-sale increase or decrease the expression of a gene, but instead disrupt co-expression or how networks of genes function together [102].

#### Post-hoc validation

Due to the exploratory nature of sequencing and the typically small sample sizes, appropriate and high-powered post hoc methods should be used to confirm findings. A popular approach is to employ qPCR, which is affordable, fast, and easily applied to a large number of samples. A complementary approach is to use methods such as *in situ* hybridization (ISH) or immunohistochemistry (IHC) to visually confirm the presence, localization, and differential expression of transcripts or proteins, respectively. While less high throughput than qPCR, these histological methods of validation have the potential to bridge ex-vivo sequencing with spatially-resolved sequencing. Popular spatial validation methods include traditional colorimetric methods with larger probes and strong contrast (e.g., alkaline phosphatase staining; [103] as well as and newer fluorescent methods with smaller probes such as MERFISH [32], RNAscope [104], and HCR [105], which allow for higher resolution and multiple target detection (multiplexing). When conducting spatial validation, it is important to consider that sites of RNA and protein synthesis are not always colocalized [106].

### Final thoughts: sometimes more is better, but sometimes more is just more

Recent advances in NGS approaches and analyses enable us to answer new questions previously out of reach with cell-type and gene-level specificity. However, if you have a specific gene or set of genes of interest that you anticipate to be robustly regulated in a disease or phenotype, do not waste money, computer power, and time with sequencing. Simply do the qPCR experiment. qPCR has served the field well for decades. Bulk-tissue and single-cell/nucleus sequencing will generate an enormous amount of data that can be fun to analyze and learn from and generate new hypotheses, but your genes of interest may or may not rise above FDR-corrected statistical thresholds given this size of the genome. It is also all too easy to analyze sequencing data endlessly, preventing crucial hypothesis-testing from moving forward. Use NGS approaches to answer specific questions as much as possible, to examine genome-wide patterns, or perhaps as “hypothesis generating” tools, but don’t be afraid to run the well-reasoned qPCR experiment just because omics tools are available.

## Data Availability

This review paper did not generate original data.
